# Genetic Cause of Hybrid Lethality Observed in Reciprocal Interspecific Crosses between *Nicotiana simulans* and *N. tabacum*

**DOI:** 10.3390/ijms25021226

**Published:** 2024-01-19

**Authors:** Takahiro Tezuka, Shota Nagai, Chihiro Matsuo, Toshiaki Okamori, Takahiro Iizuka, Wataru Marubashi

**Affiliations:** 1 Graduate School of Agriculture, Osaka Metropolitan University, Sakai 599-8531, Osaka, Japan; so22316d@st.omu.ac.jp; 2 Education and Research Field, School of Agriculture, Osaka Metropolitan University, Sakai 599-8531, Osaka, Japan; 3 Graduate School of Life and Environmental Sciences, Osaka Prefecture University, Sakai 599-8531, Osaka, Japan; t-iizuka@iskweb.co.jp; 4 School of Life and Environmental Sciences, Osaka Prefecture University, Sakai 599-8531, Osaka, Japan; 5 School of Agriculture, Meiji University, Kawasaki 214-8571, Kanagawa, Japan; marubasi@meiji.ac.jp

**Keywords:** epistasis, haplotype analysis, hybrid seedling lethality, interspecific cross, reproductive isolation, temperature sensitivity, tobacco

## Abstract

Hybrid lethality, a type of postzygotic reproductive isolation, is an obstacle to wide hybridization breeding. Here, we report the hybrid lethality that was observed in crosses between the cultivated tobacco, *Nicotiana tabacum* (section *Nicotiana*), and the wild tobacco species, *Nicotiana simulans* (section *Suaveolentes*). Reciprocal hybrid seedlings were inviable at 28 °C, and the lethality was characterized by browning of the hypocotyl and roots, suggesting that hybrid lethality is due to the interaction of nuclear genomes derived from each parental species, and not to a cytoplasmic effect. Hybrid lethality was temperature-sensitive and suppressed at 36 °C. However, when hybrid seedlings cultured at 36 °C were transferred to 28 °C, all of them showed hybrid lethality. After crossing between an *N. tabacum* monosomic line missing one copy of the Q chromosome and *N. simulans*, hybrid seedlings with or without the Q chromosome were inviable and viable, respectively. These results indicated that gene(s) on the Q chromosome are responsible for hybrid lethality and also suggested that *N. simulans* has the same allele at the *Hybrid Lethality A1* (*HLA1*) locus responsible for hybrid lethality as other species in the section *Suaveolentes*. Haplotype analysis around the *HLA1* locus suggested that there are at least six and two haplotypes containing *Hla1-1* and *hla1-2* alleles, respectively, in the section *Suaveolentes*.

## 1. Introduction

The wide hybridization between distant relatives enables the interspecific gene transfer from wild relatives into cultivated species. This method has been used for a long time and is an important tool to develop new cultivars in plant breeding [1,2,3]. However, wide hybridization breeding is often disturbed, because species are usually reproductively isolated from each other. Reproductive isolation is divided into premating, postmating–prezygotic, and postzygotic isolation barriers, and the latter two barriers are the main obstacles to wide hybridization breeding. A typical example of postmating–prezygotic barriers is the cross-incompatibility between pollen and pistils [4,5,6,7,8,9,10,11]. Postzygotic barriers include hybrid seed abortion [4,12,13,14,15,16,17,18,19,20,21,22,23,24,25,26,27,28,29,30,31,32,33,34,35,36], hybrid weaknesses [37,38,39,40,41,42,43,44,45,46,47], hybrid lethality [32,48,49,50,51,52,53,54,55,56,57,58,59,60] or necrosis [61,62,63,64,65,66,67,68,69,70,71], and hybrid sterility [72,73,74,75,76,77,78,79] in plants of the F_1_ generation, and hybrid breakdown is expressed as weaknesses, lethality, or sterility in plants of F_2_ or later generations [49,80,81,82,83,84,85,86,87,88,89]. Methods to overcome or bypass reproductive isolation are in demand for successful wide hybridization breeding [32].

The genus *Nicotiana* (Solanaceae) contains 90 species classified into 13 sections, which are predominantly distributed in the Americas and Australia [90,91]. Among *Nicotiana* species, cultivated tobacco, *N. tabacum* (2*n* = 48, SSTT), is an important cash crop. One of the main objectives of breeding programs of *N. tabacum* is to develop disease-resistant cultivars. For this purpose, wild species are useful as genetic resources, and several resistance genes, such as tobacco mosaic virus resistance gene *N* from *N. glutinosa* [92,93,94,95,96], and black shank resistance genes *Php* from *N. plumbaginifolia* and *Phl* from *N. longiflora* [97,98], have been introduced into *N. tabacum* by interspecific crossings. Another important usefulness of wild species is as a source of cytoplasmic male sterility in *N. tabacum* [99,100,101,102,103,104,105,106,107,108].

Australian wild species, *N. simulans* (2*n* = 40), which belongs to section *Suaveolentes*, is useful as a source of breeding material. This species is resistant to blue mold and powdery mildew [109,110] and is used as a source of cytoplasmic male sterile *N. tabacum* [111,112]. However, we revealed here that hybrid seedlings between *N. simulans* and *N. tabacum* showed hybrid lethality. To use the hybrid seedlings as the starting material for tobacco breeding, it is necessary to clarify the characteristics and underlying mechanism of hybrid lethality.

Section *Suaveolentes* consists of approx. 48 allotetraploid species, which are endemic to Australasia, and one allotetraploid species, *N. africana*, in Africa [91,113]. This section is monophyletic, and the species have chromosome numbers ranging from *n* = 15 to 24. After the appearance of a common ancestor at 5–6 Mya, extant species of section *Suaveolentes* are likely to have arisen through dysploid chromosome reduction [113,114,115,116,117].

Hybrid seedlings obtained from crosses between many *Suaveolentes* species and *N. tabacum* show hybrid lethality. Many of the hybrid lethality cases are caused by the epistatic interaction between the dominant allele *Hla1-1* at the *Hybrid Lethality A1* (*HLA1*) locus in *Suaveolentes* species and gene(s) on the *N. tabacum* Q chromosome, probably the dominant allele *Hla2-1* at the *HLA2* (synonym *NtHL1*) locus [56,118,119,120,121,122]. Hybrid lethality, which is called type II, is characterized by early symptoms experienced by hybrid seedlings: the browning of hypocotyl and roots. Another characteristic of type II hybrid lethality is temperature sensitivity; hybrid lethality is observed at 28 °C but suppressed at elevated temperatures ranging from 34 to 37 °C [56,121,123,124,125]. Several studies suggested that the disease resistance response is involved in type II hybrid lethality [126,127,128]. However, other hybrid lethality cases resulting from different gene combinations were also observed depending on the cross-combination [129,130]. Therefore, it is assumed that *N. simulans* has *Hla1-1*, but this needs to be verified.

A possible method to investigate whether *N. simulans* has the *Hla1-1* allele is the complementation test by triple crosses between hybrids of *N. simulans* with *Hla1-1* carriers and *N. tabacum*. However, this strategy would be hampered, because hybrids between *Suaveolentes* species are often sterile [120,131,132,133,134]. Alternatively, it is useful to characterize hybrid lethality by the phenotype and responsible chromosome. Thus, in the present study, we investigated phenotypic symptoms and the temperature sensitivity of hybrid lethality in crosses between *N. simulans* and *N. tabacum*. The *N. tabacum* chromosome responsible for hybrid lethality was identified by crossing experiments using an *N. tabacum* monosomic line for the Q chromosome. We then carried out haplotype analysis for the candidate region of the *HLA1* locus using 13 *Suaveolentes* species including *N. simulans* to determine the region containing the locus.

## 2. Results

### 2.1. Type of Hybrid Lethality Observed in Reciprocal Hybrids

Hybrid seeds were obtained from reciprocal crosses between *N. simulans* and *N. tabacum* after conventional cross-pollination (Table 1). Although only a small number of *N. tabacum* flowers was pollinated with *N. simulans*, the *N. tabacum* flowers produced few capsules (16.7% of flowers pollinated) compared with the reciprocal cross (85% of flowers pollinated); five of six flowers of *N. tabacum* dropped approximately 7 days after pollination (DAP) when pollinated with *N. simulans*. The percentage of seed germination was also different depending on the cross-direction: 68.8% when *N. simulans* was used as the female parent and 2.5% when it was used as the male parent.

**Figure 1:**
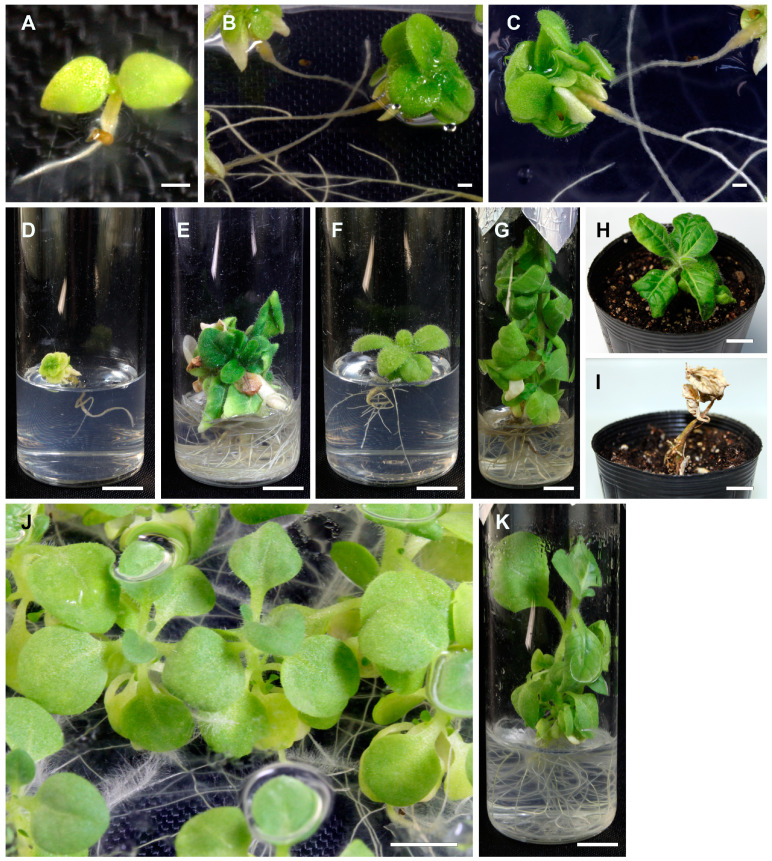
Appearances of hybrid seedlings from the cross *N. simulans* × *N. tabacum* at 28 °C (**A**–**I**) and 36 °C (**J**,**K**). (**A**) A 4 DAG hybrid seedling showing slight browning of hypocotyl. (**B**,**C**) A 33 DAG hybrid seedling photographed from the top (**B**) and bottom (**C**) of the Petri dish. The hypocotyl and base of roots turned brown. (**D**–**G**) Different degree of plant growth observed in hybrid seedlings showing lethality at 60 DAG. (**H**,**I**) A hybrid seedling at 79 and 134 DAG (**H**,**I**, respectively) after acclimatization. (**J**,**K**) Totals of 30 and 47 DAG hybrid seedlings (**J**,**K**, respectively) showing normal plant growth by suppressing hybrid lethality at 36 °C. Scale bars = 1 (**A**–**C**) or 10 mm (**D**–**K**).

All 247 hybrid seedlings obtained from reciprocal crosses were inviable at 28 °C (Table 1, Figure 1A–I). Hybrid seedlings grew normally until about 3 days after germination (DAG), but their hypocotyls turned brown in a few days, followed by the browning of roots (Figure 1A–C). These symptoms were characteristics of type II hybrid lethality. Although the degree of seedling growth varied from seedling to seedling (Figure 1D–G), all the seedlings eventually died (Figure 1H,I). Type II hybrid lethality was observed in reciprocal crosses, suggesting that hybrid lethality is due to the interaction between nuclear genomes derived from each parental species, or perhaps more exactly, genes in each nuclear genome, and not to a cytoplasmic effect.

### 2.2. Effect of Elevated Temperature on Hybrid Lethality

Twenty-five hybrid seedlings from the cross *N. simulans* × *N. tabacum* were newly obtained from 35 seeds sown in vitro at 28 °C. These seedlings were cultured at 36 °C to investigate whether hybrid lethality is suppressed at elevated temperatures. Hybrid seedlings grew normally without lethal symptoms at 36 °C (Figure 1J,K). When the hybrid seedlings were transferred from 36 °C to 28 °C at 55 DAG, they showed type II lethality and died.

### 2.3. Involvement of the Q chromosome in Hybrid Lethality

To determine whether the Q chromosome of *N. tabacum* is responsible for hybrid lethality, monosomic analysis using *N. tabacum* monosomic plants missing one copy of the Q chromosome was carried out. The monosomic plants were used as maternal parents for crossing with *N. simulans*, because the transmission of the monosomic condition through pollen is very low in *N. tabacum* [135]. Firstly, we carried out conventional cross-pollination to obtain hybrid seeds. However, 30 pollinated flowers of *N. tabacum* monosomic plants dropped approximately 7 DAP. The ovaries and ovules of these flowers did not enlarge, suggesting that fertilization did not occur. To bypass the possible prezygotic barrier, test-tube fertilization and ovule culture were carried out. Fifteen placentas of monosomic plants were pollinated with *N. simulans* pollen, resulting in 341 enlarged ovules. After ovule culture, 50 hybrid seedlings were obtained and cultured at 36 °C to suppress hybrid lethality.

Thirty-six hybrid seedlings were assessed for the presence or absence of the Q chromosome using four Q-chromosome-specific sequence tagged site (STS) markers [118,136] (Table 2). All the markers were detected in 6 hybrid seedlings but not in 30 hybrid seedlings. When the hybrid seedlings were transferred from 36 °C to 28 °C, all seedlings possessing the Q chromosome died, whereas all seedlings lacking the Q chromosome survived without lethal symptoms.

### 2.4. Haplotype Analysis of the HLA1 Candidate Region

The Q chromosome was found to be responsible for type II hybrid lethality in crosses between *N. simulans* and *N. tabacum* as that in the cross between *N. forsteri* (synonym *N. debneyi*) and *N. tabacum*. Because *N. simulans* and *N. forsteri* are closely related species, both belonging to the monophyletic section *Suaveolentes*, *N. simulans* would have the *Hla1-1* allele at the *HLA1* locus, which was originally identified in *N. forsteri* [120]. This enabled us to investigate the candidate region of the *HLA1* locus in *Suaveolentes* species by haplotype analysis.

The *HLA1* locus was mapped between two cleaved amplified polymorphic sequence (CAPS) markers, Nb14-CAPS and NbRGH1-CAPS, using the F_2_ population derived from the cross *N. forsteri* × *N. fragrans* [137]. These markers were located in the Niben101Scf06736 scaffold in the *N. benthamiana* v1.0.1 genome [138]. In the present study, we developed three new CAPS markers showing polymorphism between *N. forsteri* and *N. fragrans* based on the scaffold (Supplemental Table S1). Genotypes of all the five markers in 13 *Suaveolentes* species were investigated (Table 3). Eight haplotypes were recognized in this region, although the exact haplotype of *N. megalosiphon* could not be determined, due to the heterozygosity. Haplotypes Hap1–6 were observed in species with the *Hla1-1* allele, and Hap3 was the most common haplotype. In species with the *hla1-2* allele, two haplotypes (Hap7 and Hap8) were observed. A linkage disequilibrium was observed between Nb49-CAPS and NbRGH1-CAPS, suggesting that the *HLA1* locus is located in this region. However, the marker genotypes of Nb14-CAPS also matched well with the *HLA1* genotypes.

## 3. Discussion

For breeding purposes, it is important to clarify the underlying mechanism of hybrid lethality. This allows us to consider whether to use existing methods or develop new methods to overcome hybrid lethality. Hybrid lethality, which is caused by the epistatic interaction of the *Hla1-1* allele from *Suaveolentes* species and the *Hla2-1* allele from *N. tabacum*, is characterized by its lethal symptoms observed as the browning of hypocotyl and roots as well as temperature sensitivity [56,121,123,124,125]. All these characteristics were observed in crosses between *N. simulans* and *N. tabacum*. Furthermore, as well as crosses between other *Suaveolentes* species and *N. tabacum* [56,118,119,121], the Q chromosome containing the *HLA2* locus from *N. tabacum* was involved in hybrid lethality in crosses between *N. simulans* and *N. tabacum*. Altogether, these results indicated that *N. simulans* has the *Hla1-1* allele.

Previous studies have suggested that the disease resistance response is related to the type II hybrid lethality caused by *Hla1-1* and *Hla2-1* alleles. The involvement of several disease-resistance-related genes has been reported in crosses of *N. tabacum* with *N. gossei* (*Hla1-1*) and *N. suaveolens* (*Hla1-1*) [125,126,128]. Features of programmed cell death, which are similar to the hypersensitive response, a type of programmed cell death associated with the plant defense response, were observed in crosses of *N. tabacum* with *N. forsteri*, *N. gossei*, and *N. suaveolens* [124,139,140]. Furthermore, the *Hla2-1* allele in *N. tabacum* encodes a coiled-coil nucleotide-binding site-leucine-rich repeat (CC-NBS-LRR) protein, which might be involved in disease resistance. Considering that hybrid seedlings of the cross *N. simulans* × *N. tabacum* exhibited the same lethal symptoms and temperature sensitivity as those derived from crosses between other *Suaveolentes* species and *N. tabacum*, downstream factors of hybrid lethality triggered by *Hla1-1* and *Hla2-1* alleles would be conserved among *N. simulans* and other *Suaveolentes* species.

Although several methods to overcome hybrid lethality have been developed in the genus *Nicotiana*, their effectiveness varies depending on the type of hybrid lethality [123]. Therefore, the demonstration that *N. simulans* has the *Hla1-1* allele has great significance for overcoming hybrid lethality in crosses with *N. tabacum*. Strong similarities in hybrid lethality suggest that several overcoming methods developed for other cross-combinations of *Suaveolentes* species and *N. tabacum* may be applicable to crosses between *N. simulans* and *N. tabacum*. Tissue cultures using cotyledons of hybrid seedlings before showing lethal symptoms are effective at overcoming hybrid lethality in crosses of *N. tabacum* with *N. forsteri*, *N. rosulata*, and *N. suaveolens* [141,142]. Viable hybrids can be also obtained by the application of cytokinin to hybrid seeds or seedlings in the cross *N. suaveolens* × *N. tabacum* [143,144]. In the cross *N. gossei* × *N. tabacum*, viable hybrids are obtained by using *N. tabacum* pollen irradiated with γ-rays or ion beams for the cross [145]. By using these methods, it would be possible to obtain viable hybrids from crosses between *N. simulans* and *N. tabacum*, which can be used as the starting material for breeding.

The *HLA1* locus was mapped between markers Nb14-CAPS and NbRGH1-CAPS within a 21.7 cM genetic distance [137]. Considering that many *Suaveolentes* species have the *Hla1-1* allele, these species might have the same haplotype in the candidate region of the *HLA1* locus. Haplotype analysis in the present study suggested that the *HLA1* locus is located in the region including Nb14-CAPS or the region including Nb49-CAPS and NbRGH1-CAPS. These results provide useful information for future positional cloning efforts. Elucidating the function of the *HLA1* gene will contribute to understanding and overcoming hybrid lethality.

## 4. Materials and Methods

### 4.1. Plant Materials

*Nicotiana tabacum* (2*n* = 48, SSTT) ‘Red Russian’ was used as the parent for reciprocal crosses with *N. simulans* (2*n* = 40). We also used monosomic plants (2*n* = 47) for the Q chromosome of *N. tabacum*, which can be readily identified among F_1_ progeny obtained from the cross *N. tabacum* Haplo-Q (2*n* = 47; a monosomic line for the Q chromosome) × *N. tabacum* ‘Samsun NN’ using Q-chromosome-specific DNA markers [136]. For marker analysis of the *HLA1* candidate region, we used 12 additional *Suaveolentes* species: *N. africana*, *N. benthamiana*, *N. forsteri*, *N. excelsior*, *N. fragrans*, *N. goodspeedii*, *N. gossei*, *N. ingulba*, *N. maritima*, *N. megalosiphon*, *N. suaveolens,* and *N. velutina*. All plants were cultivated in a greenhouse under natural day length, and fertigated at each watering with Otsuka-A nutrient solution (OAT Agrio Co., Tokyo, Japan).

### 4.2. Interspecific Crosses

Conventional crossing and sowing were carried out as follows: flowers of plants used as maternal parents were emasculated one day before anthesis and pollinated with the pollen of paternal parents. F_1_ seeds were sterilized with 5% sodium hypochlorite for 15 min. The sterilized seeds were sown in Petri dishes (90 mm diameter, 17 mm depth) containing 25 mL of 1/2 MS medium [146] supplemented with 1% sucrose and solidified with 0.2% Gelrite (pH 5.8), and then cultured at 28 °C under continuous illumination (approximately 150 µmol m^−2^ s^−1^). We investigated the number of capsules obtained after crosses and seed germination rates to evaluate the presence or absence of reproductive barriers.

Test-tube fertilization in combination with ovule culture was performed as previously described [147] to obtain hybrid seedlings between *N. tabacum* Q-chromosome monosomic plants (♀) and *N. simulans* (♂). Anthers of *N. simulans* plants were aseptically excised from still-closed flowers and stimulated to dehisce in an incubator held at 28 °C. Flowers of monosomic plants were emasculated one day before anthesis. On the next day, flowers of monosomic plants were collected and their corolla, sepals, and styles were removed. The ovaries were surface-sterilized with 70% ethanol for 30 s followed by a 5% sodium hypochlorite solution for 5 min. The ovary walls were peeled back to expose the placentas with intact ovules and the ovaries were then placed in Petri dishes (60 mm diameter, 17 mm depth) containing 8 mL of medium supplemented with 3% sucrose and solidified with 0.8% agar (pH 5.8). Pollen of *N. simulans* was spread on the surface of the placentas, which were then maintained at 28 °C under continuous illumination. Fertilized and enlarged ovules were excised from placentas 10 to 14 DAP and cultured in Petri dishes (60 mm diameter, 17 mm depth) containing 8 mL of 1/2 MS medium supplemented with 3% sucrose and solidified with 0.8% agar (pH 5.8) at 28 °C under continuous illumination.

### 4.3. Cultivation of Hybrid Seedlings

Hybrid seedlings obtained from reciprocal crosses between *N. simulans* and *N. tabacum* by conventional crossing were cultured at 28 or 36 °C under continuous illumination. At 30 DAG, these seedlings were subcultured into flat-bottomed test tubes (30 mm diameter, 120 mm length) that contained 25 mL of 1/2 MS medium supplemented with 1% sucrose and solidified with 0.2% Gelrite (pH 5.8). Then, the seedlings were subcultured to fresh medium every three weeks. Seedlings cultured at 36 °C for 55 DAG were transferred to 28 °C under continuous illumination to investigate whether these seedlings showed hybrid lethality. Finally, seedlings cultured at 28 °C for 72 DAG and those cultured at 28 °C for 35 days after transfer from 36 °C (90 DAG) were transplanted to pots filled with a 3:1 (*v*/*v*) mixture of peat-moss (Super Cell-Top V; Sakata Seed Co., Yokohama, Japan) and vermiculite (Nittai Co., Osaka, Japan) and cultivated at 28 °C. Seedlings were fertigated at each watering with Otsuka-A nutrient solution (OAT Agrio Co., Tokyo, Japan).

Hybrid seedlings obtained from the cross between *N. tabacum* Q-chromosome monosomic plants and *N. simulans* by test-tube fertilization with ovule culture were transferred to flat-bottomed test tubes (30 mm diameter, 120 mm length) that contained 25 mL of 1/2 MS medium supplemented with 1% sucrose and solidified with 0.2% Gelrite (pH 5.8) immediately after germination and cultured at 36 °C under continuous illumination. The seedlings were subcultured to fresh medium every three weeks. After analyses using Q-chromosome-specific DNA markers, the seedlings were transferred to 28 °C under continuous illumination.

### 4.4. Detection of Q-Chromosome-Specific DNA Markers

Total DNA was extracted from the leaves of each plant from the cross between *N. tabacum* Q-chromosome monosomic plants and *N. simulans* using a cetyltrimethylammonium bromide (CTAB)-based method [148]. Four Q-chromosome-specific STS markers, QCS1, QCS2, QCS3, and QCS4 [118,136], were detected by conventional PCR as follows. Reaction mixtures consisted of 1× Standard Buffer (BioAcademia, Suita, Japan), 0.2 mM of each dNTP, 0.2 µM of each primer, 20 ng of template DNA, and 0.5 U of *Taq* DNA polymerase (BioAcademia) in a total volume of 20 µL. PCR amplification was performed using the TProfessional Basic Thermocycler (Biometra, Göttingen, Germany) programmed for 3 min at 94 °C for initial denaturation, followed by 35 cycles of 30 s at 94 °C, 30 s at 60 °C, and 30–90 s at 72 °C, with a final 5 min extension at 72 °C. PCR products were separated by electrophoresis in 1.5% agarose gels with TBE buffer and were then visualized by staining with ethidium bromide.

### 4.5. Marker Analysis for the HLA1 Candidate Region

Total DNA was extracted from the young leaves of each *Nicotiana* species using a CTAB-based method [148]. Two CAPS markers linked to *HLA1*, Nb14-CAPS and NbRGH1-CAPS, were detected as previously described [137]. In the *HLA1* candidate region between these markers, three new CAPS markers, Nb45-CAPS, Nb48-CAPS, and Nb49-CAPS (Table S1), were developed based on the sequence of Niben101Scf06736 scaffold in the v1.0.1 draft genome sequence of *N. benthamiana* [138], and detected as in the previous study [137].

## 5. Conclusions

Reciprocal hybrid seedlings from crosses between *N. simulans* and *N. tabacum* exhibited type II hybrid lethality characterized by browning of the hypocotyl and roots, suggesting that hybrid lethality is due to the interaction of nuclear genomes derived from each parental species, and not to a cytoplasmic effect. Hybrid lethality was temperature-sensitive and suppressed at 36 °C. Furthermore, the Q chromosome containing the *HLA2* locus from *N. tabacum* was revealed to be involved in hybrid lethality. Altogether, these results indicated that *N. simulans* has the *Hla1-1* allele, the same allele at the *HLA1* locus as many other species in the *Nicotiana* section *Suaveolentes*. Haplotype analysis around the *HLA1* locus suggested that there are at least six and two haplotypes containing *Hla1-1* and *hla1-2* alleles, respectively, in the section *Suaveolentes*. Elucidating the function of the *HLA1* gene through positional cloning efforts will contribute to understanding and overcoming hybrid lethality.

## Figures and Tables

**Table 1:** Viability of reciprocal hybrids between *N. simulans* and *N. tabacum* at 28 °C.

Cross Combination (♀ × ♂)	No. of Flowers Pollinated	No. of Capsules Obtained	No. of Seeds Sown	No. of Hybrids Obtained			Lethality Type ^1^
				Total	Viable	Inviable	
*N. simulans* × *N. tabacum*	20	17	352	242	0	242	II
*N. tabacum* × *N. simulans*	6	1	199	5	0	5	II

^1^ Type II, browning of hypocotyl and roots.

**Table 2:** Relationship between the Q chromosome and hybrid lethality in crosses between *N. tabacum* and *N. simulans*.

Cross Combination (♀ × ♂)	STS Markers ^1^	No. of Hybrids		
		Total	Viable	Inviable
(Haplo-Q × ‘Samsun NN’) × *N. simulans*	+	6	0	6
	−	30	30	0

^1^ ‘+’ indicates that Q-chromosome-specific STS markers were detected and ‘−’ indicates that they were not.

**Table 3:** Haplotypes identified in the *HLA1* candidate region.

Haplotype	Species	Allele at the *HLA1* Locus	Marker				
			Nb14-CAPS (150 kb) ^1^	Nb45-CAPS (624 kb)	Nb48-CAPS (725 kb)	Nb49-CAPS (747 kb)	NbRGH1-CAPS (832 kb) ^3^
Hap1	*N. forsteri*	*Hla1-1*	*AA* ^2^	*AA*	*AA*	*AA*	*AA*
Hap2	*N. ingulba*, *N. simulans*	*Hla1-1*	*AA*	*AA*	*BB*	*AA*	*AA*
Hap3	*N. excelsior*, *N. goodspeedii*, *N. gossei*, *N. maritima*, *N. velutina*	*Hla1-1*	*AA*	*BB*	*BB*	*AA*	*AA*
Hap4	*N. suaveolens*	*Hla1-1*	*AA*	*BB*	−	*AA*	*AA*
Hap5	*N. megalosiphon*	*Hla1-1*	*AB*	*AB*	−	*AA*	*AA*
Hap6	*N. africana*	*Hla1-1*	*AA*	*BB*	*CC*	*AA*	*B*
Hap7	*N. benthamiana*	*hla1-2*	*BB*	*BB*	*BB*	*AA*	*AA*
Hap8	*N. fragrans*	*hla1-2*	*BB*	*BB*	*BB*	*BB*	*B*

^1^ Value in parenthesis indicates the approximate position of the marker in the Niben101Scf06736 scaffold in the v1.0.1 genome of *N. benthamiana*. ^2^ ‘*A*’ and ‘*B*’ indicate *N. forsteri*-type and *N. fragrans*-type alleles, respectively. ‘−’ indicates no detectable band. ^3^ This marker cannot discriminate between *AB* and *BB* genotypes [137].

## Data Availability

The original contributions presented in the study are included in the article/supplementary material, further inquiries can be directed to the corresponding author.

## Supplementary Materials

The following supporting information can be downloaded at https://www.mdpi.com/article/10.3390/ijms25021226/s1.
